# Exploring the understanding of best practice approaches to common dog behaviour problems by veterinary professionals in Ireland

**DOI:** 10.1186/s13620-019-0139-3

**Published:** 2019-03-21

**Authors:** Emma Shalvey, Mark McCorry, Alison Hanlon

**Affiliations:** 1 School of Veterinary Medicine, University College Dublin, Dublin, Ireland; 2 Blacklion Pet Hospital, Greystones, County Wicklow Ireland

**Keywords:** Veterinary behaviour medicine, Veterinary education, Continuing veterinary education, Dog behaviour, Dog training, Dominance

## Abstract

**Background:**

Companion animal behaviour problems significantly impact companion animal (and owner) welfare. Veterinary behavioural medicine (VBM) is an emerging discipline and aims to provide evidence-based advice to owners and veterinary professionals to support normal behaviour in companion animals through appropriate socialisation and training and to address behaviour problems in a constructive and welfare-friendly manner. The approach to problem behaviours in dogs has changed in recent years; previously a mis-understanding of the biological theory of dominance has been used to explain certain behavioural problems in dogs which has led to the use of punishment-based treatment methods. Current research advocates the benefits of reward-based methods and highlights the risks of implementing positive punishment-based training techniques to both dogs and owners. Golden and Hanlon (Ir Vet J 71: 12, 2018) have reported that veterinary professionals in Ireland are frequently asked to advise on dog behaviour problems. This study aimed to explore veterinary professionals’ understanding of training and treatment options for frequently encountered dog behaviour problems, and to help support the development of competences in VBM in Ireland.

**Methods:**

An online survey was developed, including a pre-test evaluation by a pilot group of veterinary professionals, on SurveyMonkey®. The link to the online survey was distributed via third-party professional associations and social media. The survey contained twelve vignettes illustrating advice from veterinary professionals on common behaviour scenarios. Using a Likert Scale, respondents were asked to assess the likelihood of the advice to support best outcome for the dog. Best outcome was defined as one which provides a resolution to the behavioural problem while not compromising the animal’s welfare.

**Results:**

84 private veterinary practitioners (PVP) and 133 veterinary nurses (VN) completed the survey. In the majority of vignettes, most veterinary professionals agreed with our classification of best outcome, but several areas of uncertainty were identified. Marked variations in response were found for PVPs in vignettes depicting advice recommending citronella collars, invisible radio fences, trainers utilising dominance language, and another dog for separation anxiety. For VNs, variations in response were found in vignettes depicting dominance-based training and advice on separation anxiety. Significant differences were found in the responses of VNs and PVPs for the vignettes recommending the use of citronella collars (*p* < 0.01) and invisible radio fences (*p* < 0.05), where VNs agreed with their recommendation less often than PVPs. PVPs graduating since 2013 agreed with the recommendation of invisible radio fences less often than PVPs graduating before 2013 (*p* < 0.05). VNs graduating before 2013 agreed with the recommendation of an accredited trainer (p < 0.05) and disagreed with the use of flooding to treat fear (p < 0.05) more often than VNs graduating since 2013.

**Conclusions:**

Our findings have identified specific areas of uncertainty with regards knowledge of positive punishment-based training and the treatment of common dog behaviour problems, highlighted the demand for continuing professional education in VBM and provided further evidence of the need to develop day one competences in VBM for veterinary medicine and nursing programmes at university level.

**Electronic supplementary material:**

The online version of this article (10.1186/s13620-019-0139-3) contains supplementary material, which is available to authorized users.

## Background

The main approach to animal training and treatment of behaviour problems in dogs has changed in the last 10 years. Prior to this many considered aggressive behaviour to be derived from a dog’s desire to be ‘dominant’ and establish its place in the hierarchy (e.g. Jones-Baade & McBride [16]); this premise was applied to humans’ relationships with dogs and formed the commonly-used basis for approaching behaviour problems in dogs [15]. Dominance theory has since been called into question, as the studies used to support it observed aggressive interactions between unrelated captive wolves, with further studies on naturally-assembled wild wolf packs failing to show the same level of aggressive interactions [5]. In addition, social structures differ between packs of feral dogs and wolves, with interactions between dogs less predicted by hierarchy, suggesting that domestication has changed the social behaviour of dogs [5].

Positive punishment-based training is when an undesirable behaviour is discouraged by the addition of an aversive stimulus. The application of positive punishment-based training methods founded in dominance theory are not supported by current literature. However, there is continued use of such training techniques including physical reprimands to combat behavioural problems in dogs [4, 21]. The use of these training methods may not only be ineffective, but also unethical because of the risk of harm such as pain and fear caused to the animal [2, 6, 33].

A survey of owners [13] on the use of ‘confrontational’ positive punishment-based training methods on dogs for a variety of behaviour problems evaluated the outcome of performing direct confrontational techniques (e.g., “alpha-rolls”), and indirect confrontational techniques (e.g. “yelling no”), versus the use of non-confrontational techniques and positive reinforcement, namely, reward-based training. They found a high frequency of aggressive responses from the dogs towards confrontational techniques. This highlights the risk of positive punishment-based techniques and the important role of the private veterinary practitioner (PVP) in informing clients about the dangers of their use in the home. It is important that a PVP be informed of the options available for behaviour modification, especially when dealing with potentially dangerous behaviour problems such as aggression, so that they can adequately inform clients and promote the use of effective and humane methods (for example positive reinforcement training, desensitisation and counter conditioning) and advise against methods that are both dangerous and ineffective [33].

Whilst treatment of behaviour problems with positive punishment-based training methods can be dangerous for the owner, they can also result in poor welfare outcomes for the dog, and often with little or no improvement in the problem behaviour. The use of electronic training collars has been associated with poor welfare in dogs. Cooper et al. [7] found increased indicators of stress in dogs trained using electronic training collars and reported no benefit in using these tools over reward-based training. These devices are banned in Wales and a ban is proposed in England; yet they remain legal without restriction in the Republic of Ireland. Other more traditional positive punishment-based methods can also lead to physical harm for the animal involved [11]. Grohmann et al. reported severe brain damage in a German Shepherd dog following positive punishment-based training via the use of a check chain by its owner during a training class. In addition, the use of positive punishment-based methods in dogs with behaviour problems rooted in anxiety can worsen the behaviours and result in poor welfare outcomes for the dog. The use of positive punishment-based methods is associated with increased anxiety and fear and may lead to reinforcement of fear and fear-related undesirable behaviours, including aggression [2]. For example, punishment for destructive behaviour associated with separation anxiety is likely to be ineffective and dogs are less anxious when their training is based around positive reinforcement [27]. Therefore, whether dealing with aggression or other behaviour problems based in anxiety and fear, positive punishment-based methods do not offer any benefit over reward-based methods, and often lead to worse outcomes.

Blackwell et al. [4] found that 72% of dog owners surveyed utilised some form of positive punishment-based techniques corresponding to an increased incidence of undesirable behaviours (aggression and avoidance). The prevalence in the use of positive punishment-based training methods by owners, in addition to their associated undesired outcomes, highlights the importance that PVPs be informed in the selection of appropriate training methods, as they can result in negative outcomes and potential danger for both dog and owner [18].

Veterinary professionals frequently encounter behaviour problems in practice [10], and many owners use their PVP as a source of behaviour advice [21, 24]. Yet, research in both the UK and Ireland has highlighted that many veterinary professionals considered their education in animal behaviour to have been inadequate and that they feel ill-equipped to advise clients on animal behaviour problems [10, 25].

Current research places emphasis on reward-based methods over positive punishment-based training [9, 30], the importance of the role of veterinary professionals in directly providing, or signposting clients to services such as puppy socialisation classes, and educated and informed advice to clients seeking help with behaviour problems in their dogs [15, 30, 33]. With the persistence in some circles of dominance theory-based methods, it is imperative that PVPs be knowledgeable on best practice approaches to behaviour problems and recognise when and to whom to refer training or a behaviour problem. The aim of this survey was to explore veterinary professionals’ understanding of different approaches to training and treatment options for canine behaviour problems commonly encountered in practice, to identify areas where there exists a lack of understanding, and to help support the development of competences in veterinary behaviour medicine in Ireland.

## Methods

A survey was designed using Survey Monkey® to explore the current understanding of private veterinary practitioners (PVP) and veterinary nurses (VN) in Ireland about treatment options for frequently encountered behaviour problems in dogs. The survey was sent out for peer review to two PVPs (one with a specialist qualification in behaviour), five VNs (one with a specialist qualification in behaviour) and one non-veterinary behaviourist. Amendments were made based on their feedback before it was published on Survey Monkey.

The survey commenced on the 28th of June 2018. An invitation to complete the online survey was distributed by Veterinary Ireland Companion Animal Society (VICAS) via their E-newsletter and by the Irish Veterinary Nurses Association (IVNA). In addition, the link to the online survey was shared on social media via UCD School of Veterinary Medicine Twitter, UCD Veterinary Hospital Facebook and was also posted to the Veterinary Voices Ireland Facebook. Two reminders were sent out: on the 16th of July via social media and the IVNA, and on the 1st of August by VICAS. The survey was closed on the 7th of August 2018.

### Survey design

The survey consisted of 18 questions, divided into three sections: Professional Role and Experience, Scenarios on Common Canine Behavioural Problems and Continuing Education. The first question in Section One established consent in use of the respondent’s data for this research. Questions 2 to 4 recorded demographic information. Question 2 registered the profession of the respondent; Private Veterinary Practitioner (PVP), Veterinary Nurse (VN) or Other. Participants selecting ‘other’ were requested to specify their role and were brought to the end of the survey. Year of graduation and behavioural services offered in the practice (Puppy socialisation classes, Training events, In-house behavioural consultations, None and Other) were requested in Q3 and Q4, respectively.

Section Two presented 12 vignettes which depicted advice from a veterinary professional regarding dog behaviour and/or training (see Table 1). The vignettes were designed to illustrate common scenarios that are either likely (Q 6, 9, 11) or unlikely to support the best outcome for the dog (Q 5, 7, 8, 10, 12, 13, 14, 15, 16). Initially vignettes were designed to have equal examples of advice unlikely and likely to give the best outcome, but following peer review this was amended as it became clear that advice likely to give the best outcome was easily identified by peer reviewers. Behaviour problems used in each vignette were selected based on recent research by Golden and Hanlon [10] and current literature identifying commonly encountered behaviour problems [21]. Respondents were asked to select whether the advice offered by the veterinary professional in each vignette would give the best outcome, using a Likert scale (Extremely Likely, Likely, Neither Likely nor Unlikely, Unlikely, Extremely Unlikely, Don’t Know). The best outcome was defined at the start of the survey as one which provides a resolution to the behavioural problem while not compromising the animal’s welfare.

**Table 1:** Theme and contents of 12 peer reviewed vignettes depicting advice from a veterinary professional regarding dog behaviour and/or training. Vignettes were designed to illustrate common scenarios that are either likely or unlikely to support best outcome for the dog. The best outcome was defined as one which provides a resolution to the behavioural problem while not compromising the animal’s welfare

Vignette Theme	Vignette	Evidence-base for likelihood to achieve best outcome
1. Use of physical correction to treat unruly behaviour	Sarah has brought in her 1-year-old Labrador cross Toby to the vet for his annual check-up. She asks how to stop Toby from jumping up and mouthing, “*He’s knocked the kids over several times!*” The vet tells Sarah, “*Try pushing Toby down and saying ‘STOP’ when he jumps up and mouths, to discourage the behaviour”*.	Unlikely [14]
2. Use of positive reinforcement in toilet training	Paul has brought in his 8-week-old puppy for its vaccinations. He asks Sinead, the vet nurse, how to toilet train his new puppy. Sinead advises, “*Take the puppy outside at regular intervals and praise him whenever he toilets outside and don’t punish him if he has an accident inside – but make sure to clean it up properly!*” She gives Paul a leaflet on the “do’s and don’ts” of toilet training a new puppy.	Likely [14]
3. Use of citronella spray collar to treat barking	The neighbours have complained about Louise’s two dogs that bark excessively while she is at work. While buying food at the vets, she asks Lauri, the vet nurse, for advice. Lauri suggests using anti-bark spray collars, “*They give the dog a warning beep before spraying citronella, they don’t harm the dog at all”.*	Unlikely [34, 26]
4. Use of physical restraint to treat fear during nail clipping	Jack’s dog Monty is terrified of getting his nails clipped. He asks Val, a vet nurse, for advice to reduce Monty’s fear. Val advises, “*No dog likes getting their nails done, you’ve just got to restrain them and push through or else they will learn to get away with it*”.	Unlikely [4]
5. Recommendation of reward-based accredited trainer for dog reactivity	Stephen’s 4-year-old Husky lunges, growls and barks at other dogs in the vet clinic waiting room. Suzy, a vet nurse, notices the difficulty Stephen is having with controlling his dog and offers Stephen a business card, “*Several clients have had help from one of our registered APDT Ireland*^*[1]*^ *trainers*”. [1] APDT Ireland = Association of Pet Dog Trainers Ireland	Likely [14]
6. Positively reinforcing fear behaviour	Lucy has brought her 5-month-old puppy in to the vets for a quick weigh-in. Lucy asks the vet nurse, Chris, if he has any recommendations to help with her puppy’s fear during fireworks, “*She hides behind the couch all night when they’re going”* Chris offers her advice “*Give her lots of cuddles and praise when she’s feeling scared to help her feel more comfortable*”.	Unlikely [29]
7. Recommendation of obedience classes and positive reinforcement for recall training	George has brought in his Jack Russell Terrier, Skip, for a check-up following surgery after a road traffic accident. Shannon, the vet, gives Skip the all clear, but George is worried about letting him off lead again because Skip normally runs off and ignores his calls. Shannon suggests a local obedience class, “*Give these classes a try and keep Skip on a long lead during walks until you’re comfortable he’ll come back to you. If you regularly call him during your walks and reward him for coming back he’ll start to get the idea”.*	Likely [14]
8. Use of invisible radio fence to prevent wandering	John’s German Shepherd, Max, is repeatedly escaping from the garden. There have been recent cases of sheep worrying in the area and he’s looking for advice from the local practice. The vet suggests, “*It depends on how much time and money you are willing to invest – the quickest way is to install an invisible radio fence*”.	Unlikely [22, 28, 31]
9. Recommendation of trainer advocating dominance/pack theory	During a routine clinical examination, Greta’s Border Collie cross Lulu snaps at the vet. Greta admits that Lulu can be aggressive, especially towards strangers whilst on walks. The vet recommends a local trainer and tells her, “*This guy knows his stuff and will set her straight, she needs to learn you’re in charge otherwise she will keep trying to protect you and hurt other people*”.	Unlikely [5]
10. Use of check chain to treat pulling on the lead	On arrival at the vet clinic, Julie almost falls over as Ben, her Saint Bernard, pulls her through the door. The vet nurse at the desk sees that Julie is having problems controlling Ben and says, “*Have you considered using a check chain as a training aid? It’s the best way to control a big dog like Ben*”.	Unlikely [14, 11]
11. Use of desensitisation to treat fear behaviour	Tom has brought his new puppy, Penny, to the vets for her first vaccinations, “*She’s terrified of the kids at home and just cowers in the corner”* The vet replies, *“Get as many kids in from the neighbourhood as possible to handle her, that should get her well socialised”*	Unlikely [8]
12. Recommendation of acquiring another dog to treat separation anxiety	Emily has brought her lurcher, Finn, to the vet to get treatment for an injured paw after he attempted to escape from his crate “*I feel awful, but I have to confine him in there when I’m at work otherwise he destroys the house, he gets so distressed when I leave”* Fran, the vet, has seen this problem many times and suggests, *“Have you considered getting another dog to keep Finn company?”*	Unlikely ([20, 12])

Questions 17 and 18 focused on continuing veterinary education (CVE) in veterinary behaviour medicine. Respondents were asked whether they would like to learn more about veterinary behavioural medicine and the format of CVE they would like to see offered in the future. A copy of the survey can be found in the additional files (see Additional file 1). 

### Data analysis

Data was exported from SurveyMonkey® into Microsoft Excel (2016). R version 3.5.1 [23] was used for data cleaning and transformation, data visualisation, generating descriptive statistics and for all statistical analyses. Microsoft Excel (2016) was also used for generating descriptive statistics in some instances. To test for statistically significant differences in the distributions of responses between independent cohorts, a Wilcoxon rank sum test was used. ‘Don’t Know’ data were excluded for the purposes of these analyses, because the Wilcoxon rank sum test requires ordinal data. In addition, the Likert scale responses were pooled for extremely likely and likely, and extremely unlikely and unlikely, providing three categories for analyses: ‘Unlikely’, ‘Neither Likely nor Unlikely’ and ‘Likely’. Comparisons were made between the responses of PVPs and VNs, PVPs who graduated since 2013 and PVPs who graduated before 2013, VNs who graduated since 2013 and VNs who graduated before 2013, and between respondents who worked at practices where no behavioural services were offered and where behavioural services were offered. The rationale for selecting 2013 was due to the change to the VN course, which was first offered as a four-year honours Bachelor of Science in UCD in 2009, with first graduates in 2013. In addition, the changing perspective on dominance theory in dogs highlighted by Bradshaw et al. [5] was introduced into the animal behaviour and welfare module of the second year preclinical veterinary medicine programme in 2010 (students graduating in 2013). The significance threshold for statistical analyses was *P* < 0.05.

## Results

**Fig. 1:**
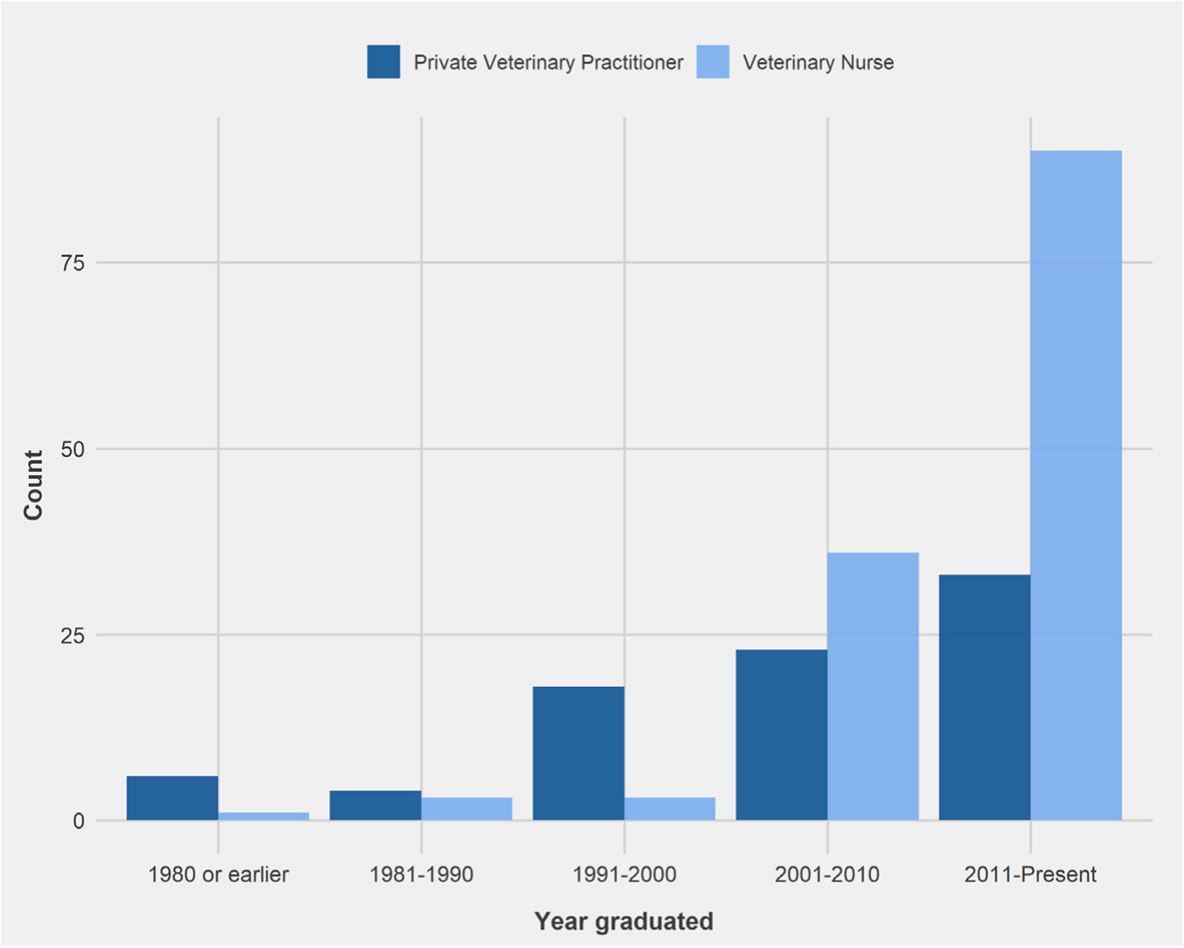
Year of graduation of Private Veterinary Practitioner (*n* = 84) and Veterinary Nurse (*n* = 133) respondents. PVP: Private Veterinary Practitioner. VN: Veterinary Nurse

Due to the method of distribution of this survey, an accurate response rate could not be calculated. A total of 338 individuals accessed the survey and 217 (64.2%) individuals completed it, comprising 84 PVPs (38.7%) and 133 VNs (61.3%). Thirty seven (12.8%) participants selected ‘Other’ and were excluded from the survey. They included veterinary students, veterinary academics, dog trainers, dog breeders, and animal rescue workers. The average time to complete the survey on SurveyMonkey was 10 min. The majority of PVP and VN respondents graduated between 2011 and 2018 (Fig. 1).

### Behavioural services offered in veterinary practice

**Fig. 2:**
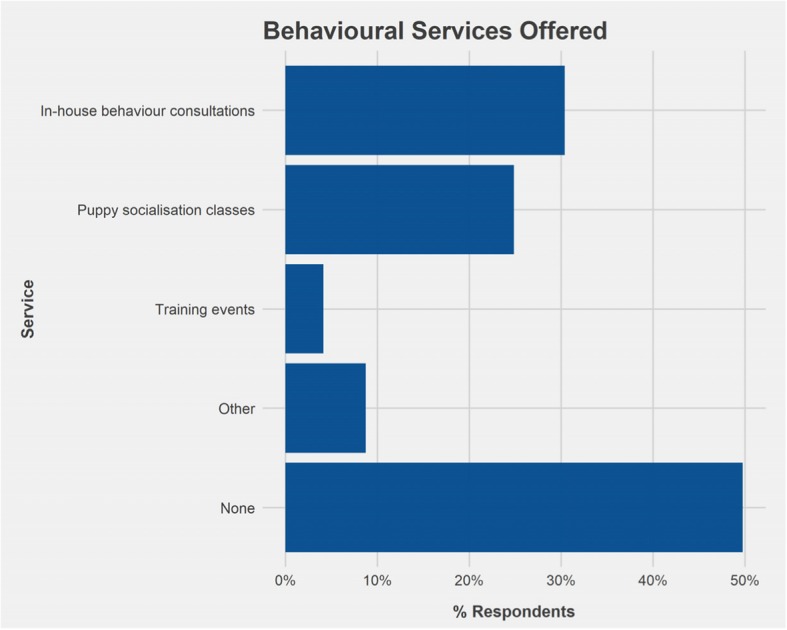
Behavioural services offered at practices in which respondents were employed. Respondents could select multiple responses (*n* = 217)

A similar percentage of respondents worked in a practice that either offered at least one form of behaviour service (46.1%), including in-house behaviour consultations, puppy socialisation classes and training events (Fig. 2), or offered no behavioural services (49.8%). In addition, 4.1% of respondents worked in a practice that provided only ‘other behaviour services’. Respondents were requested to provide information on the latter and examples included consultation with a behaviourist that visits the practice, referral to veterinary behaviourists and trainers.

### Likelihood of achieving best outcome

**Fig. 3:**
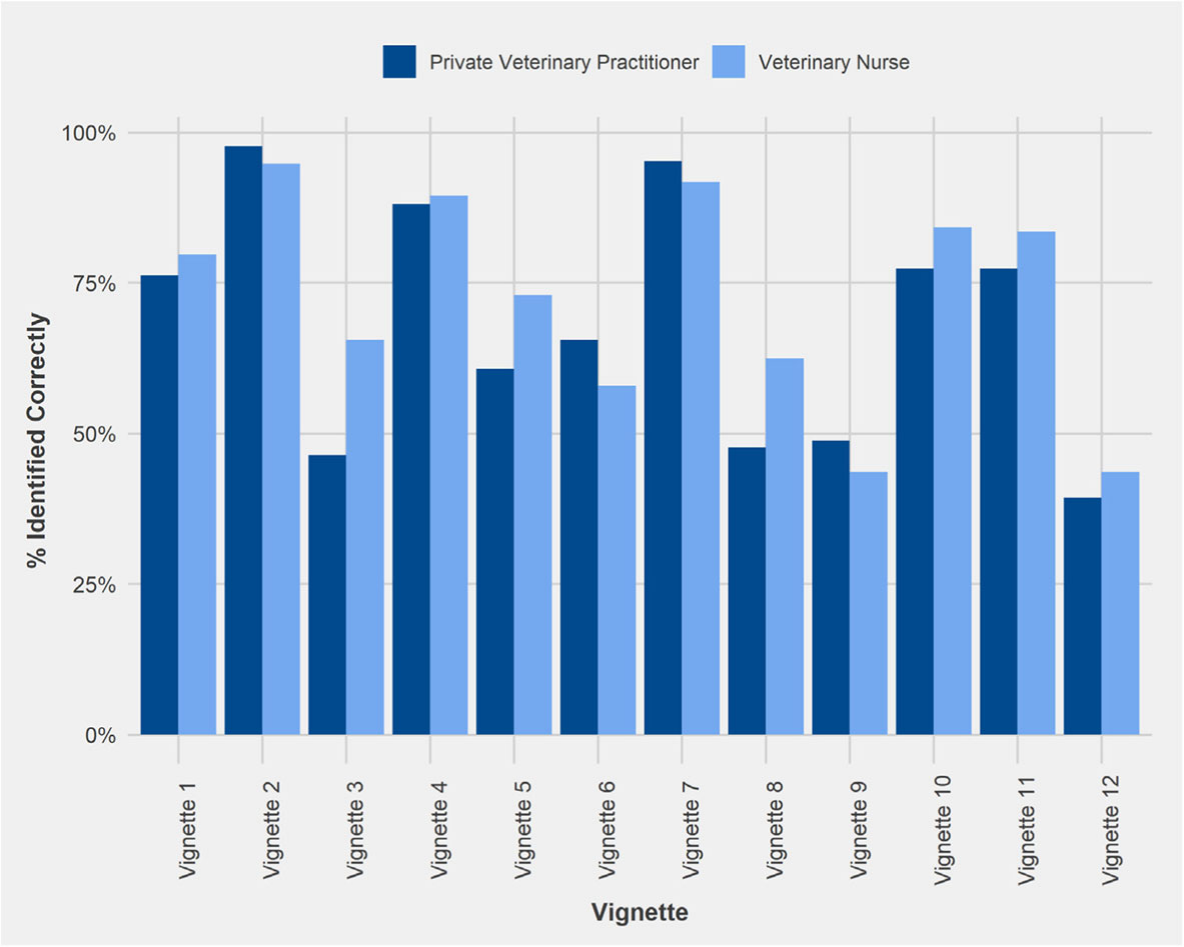
Percentage of Private Veterinary Practitioners (n = 84) and Veterinary Nurses (n = 133) who identified likelihood of advice to support best outcome for the dog for each vignette. Vignette 1 = physical correction, 2 = positive reinforcement for toilet training, 3 = citronella collar, 4 = physical restraint, 5 = accredited trainer, 6 = positively reinforcing fear, 7 = positive reinforcement for recall training, 8 = invisible radio fence, 9 = dominance trainer, 10 = check chain, 11 = desensitisation, 12 = another dog for separation anxiety (See Table 1 for full details)

The percentage of respondents who correctly identified whether advice was likely to support the best outcome is illustrated in Fig. 3. There were four vignettes where less than 50% of PVPs identified best outcome; vignettes 3 (citronella collar; 46%), 8 (invisible radio fence; 48%), 9 (dominance trainer; 49%), and 12 (another dog for separation anxiety; 39%). For VNs only two vignettes received < 50% recognition of best outcome; vignettes 9 and 12. The vignette with highest consensus amongst PVPs and VNs on achieving the best outcome was vignette 2 (positive reinforcement in toilet training; 98 and 95% respectively).

**Fig. 4:**
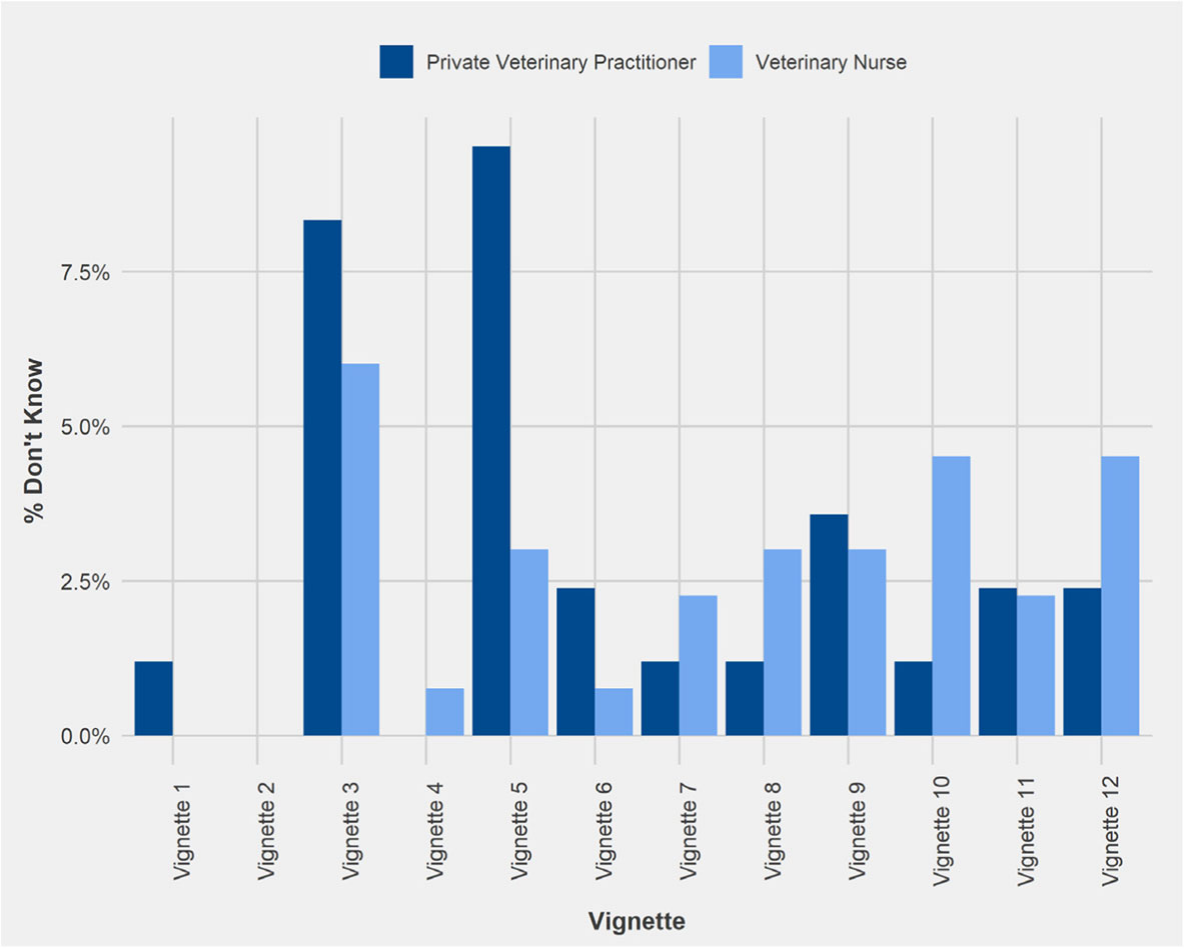
Percentage of Private Veterinary Practitioners (n = 84) and Veterinary Nurses (n = 133) respondents who answered ‘Don’t Know’ regarding the likelihood of advice to support best outcome for the dog in each vignette. Vignette 1 = physical correction, 2 = positive reinforcement for toilet training, 3 = citronella collar, 4 = physical restraint, 5 = accredited trainer, 6 = positively reinforcing fear, 7 = positive reinforcement for recall training, 8 = invisible radio fence, 9 = dominance trainer, 10 = check chain, 11 = desensitisation, 12 = another dog for separation anxiety (See Table 1 for full details)

Areas of uncertainty with best outcome were recorded for vignette 12 with only 39 and 44% of PVPs and VNs respectively, correctly identifying that the advice was unlikely to lead to the best outcome. Likewise, for vignette 9 only 44% of VNs correctly identified that the advice was unlikely to achieve best outcome.

A Wilcoxon rank sum test revealed that the percentage of respondents correctly identifying best outcome scenarios (vignettes 2, 5 and 7) was significantly higher than the percentage recognising poor likelihood of best outcome (vignettes 1, 3, 4, 6, 8, 9, 10, 11, 12) for both PVPs (W = 5369, *p* < 0.01) and VNs (W = 13,546, p < 0.01). However, there was no significant difference between the PVPs and VNs recognising likely or unlikely best outcomes.

The vignette which showed the least agreement among respondents’ answers overall was vignette 12 (separation anxiety) with a combined PVP and VN consensus of 42% (Table 2) and the vignette with the highest consensus overall (96%) was vignette 2 (toilet training; Table 2). In all cases the most frequent answer aligned with correct identification of likely or unlikely best outcome.

**Table 2:** Table illustrating the most frequent response of Private Veterinary Practitioners (PVPs), Veterinary Nurses (VNs) and the overall most frequent response (PVPs and VNs combined) in each vignette. The Best Outcome column indicates whether the advice in the given vignette was likely or unlikely to result in best outcome. Higher % indicates greater agreement on the likelihood of best outcome depicted in the vignette, while lower % indicates a more varied response. Vignette 1 = physical correction, 2 = positive reinforcement for toilet training, 3 = citronella collar, 4 = physical restraint, 5 = accredited trainer, 6 = positively reinforcing fear, 7 = positive reinforcement for recall training, 8 = invisible radio fence, 9 = dominance trainer, 10 = check chain, 11 = desensitisation, 12 = another dog for separation anxiety (See Table 1 for full details)

Vignette	Best Outcome	PVP Most Frequent Response	VN Most Frequent Response	Overall Most Frequent Response
1	Unlikely	76.2% Unlikely	79.7% Unlikely	78.3% Unlikely
2	Likely	97.6% Likely	94.7% Likely	95.9% Likely
3	Unlikely	46.4% Unlikely	65.4% Unlikely	58.1% Unlikely
4	Unlikely	88.1% Unlikely	89.5% Unlikely	88.9% Unlikely
5	Likely	60.7% Likely	72.9% Likely	68.2% Likely
6	Unlikely	65.5% Unlikely	57.9% Unlikely	60.8% Unlikely
7	Likely	95.2% Likely	91.7% Likely	93.1% Likely
8	Unlikely	47.6% Unlikely	62.4% Unlikely	56.7% Unlikely
9	Unlikely	48.8% Unlikely	43.6% Unlikely	45.6% Unlikely
10	Unlikely	77.4% Unlikely	84.2% Unlikely	81.6% Unlikely
11	Unlikely	77.4% Unlikely	83.5% Unlikely	81.1% Unlikely
12	Unlikely	39.3% Unlikely	43.6% Unlikely	41.9% Unlikely

The percentage of respondents who answered ‘Don’t Know’ for each vignette ranged from 0 to 10% (Fig. 4). The highest percentage of PVPs who answered ‘Don’t Know’ was recorded for vignette 5: ‘accredited trainer’ (10%), whereas the highest percentage of VNs who answered ‘Don’t Know’ was for vignette 3: ‘citronella collar’ (6%). No PVPs answered ‘Don’t Know’ for vignette 2: ‘positive reinforcement for toilet training’ or 4: ‘physical restraint’, whereas no VNs answered ‘Don’t Know’ for vignettes 1 (‘physical correction’) and 2. The greatest contrast in percentage of respondents who answered ‘Don’t Know’ was found in vignette 5, where 10% of PVPs answered ‘Don’t Know’ compared with only 3% of VNs.

### Comparison by profession

**Fig. 5:**
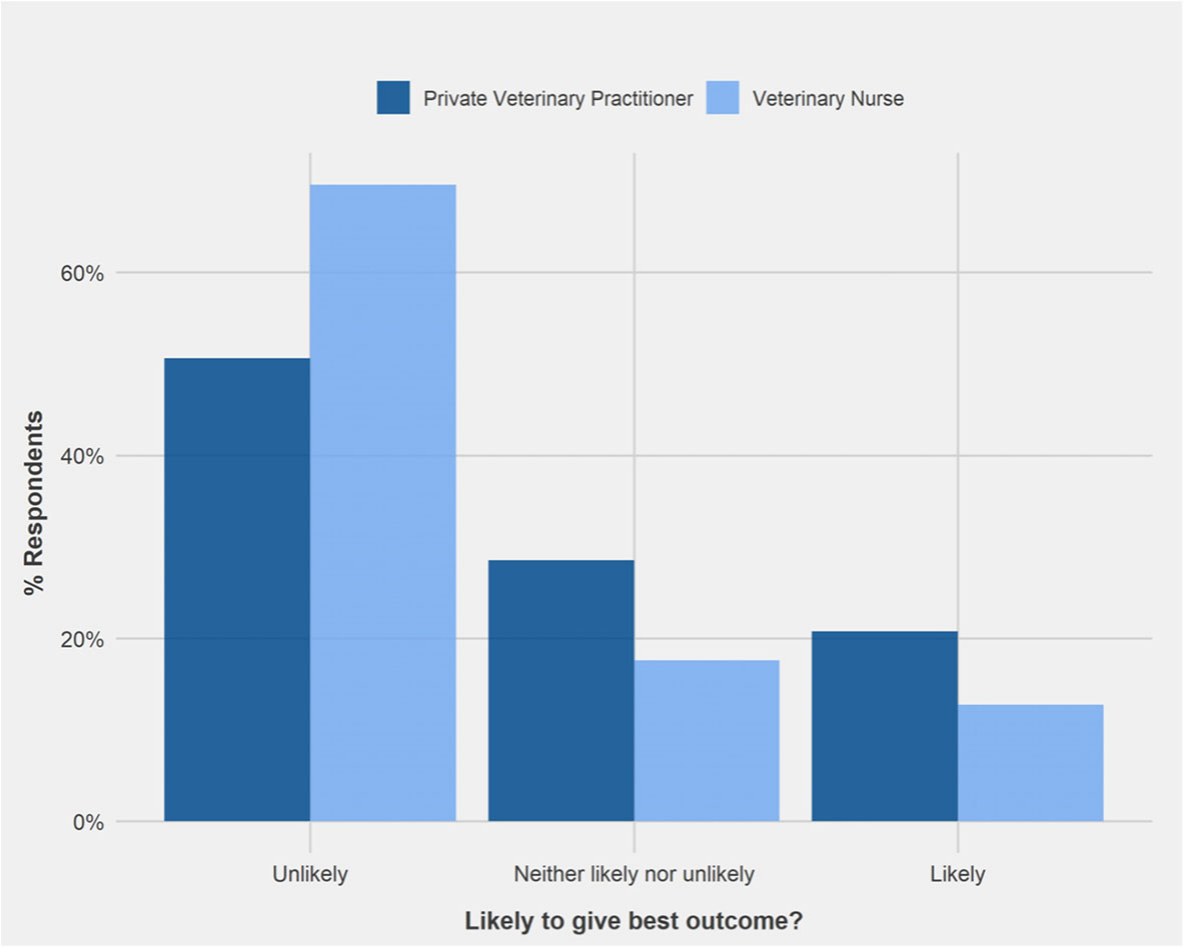
Comparison of the responses of Private Veterinary Practitioners (*n* = 77) and Veterinary Nurses (*n* = 125) regarding the likelihood of advice to support best outcome for the dog in Vignette 3: ‘Use of citronella spray collar to treat barking’

**Fig. 6:**
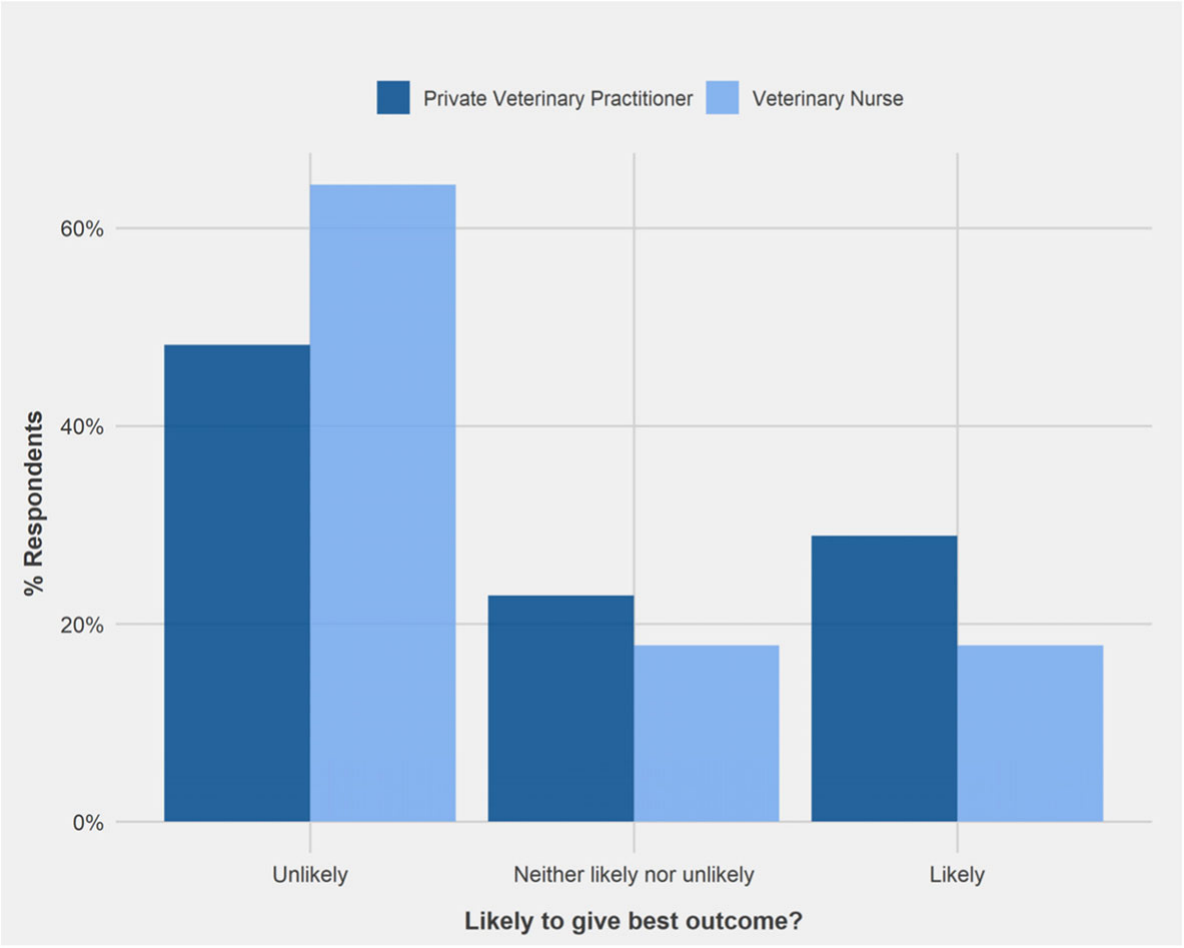
Comparison of the responses of Private Veterinary Practitioners (*n* = 83) and Veterinary Nurses (*n* = 129) regarding the likelihood of advice to support best outcome for the dog in Vignette 8: ‘Use of invisible radio fence to prevent wandering’

A Wilcoxon rank sum test showed that there was a significant difference in the distribution of responses of PVPs and VNs to vignette 3: ‘citronella collar’ (W = 6627.5, *p* < 0.01) and vignette 8: ‘invisible radio fence’ (W = 6406.5, *p* < 0.05). A smaller percentage of VNs (12.8%) than PVPs (20.8%) indicated that the advice was likely to achieve best outcome in vignette 3, while 69.6% of VNs considered that it was unlikely compared with 50.6% of PVPs (Fig. 5). In contrast 28.9% of PVPs considered that advice on using an invisible fence (vignette 8) was likely to achieve best outcome for the dog, compared to 17.8% of VNs. For this scenario 48% of PVPs and 64% of VNs correctly said it was unlikely to support the best outcome (Fig. 6).

### Comparison by graduation date

**Fig. 7:**
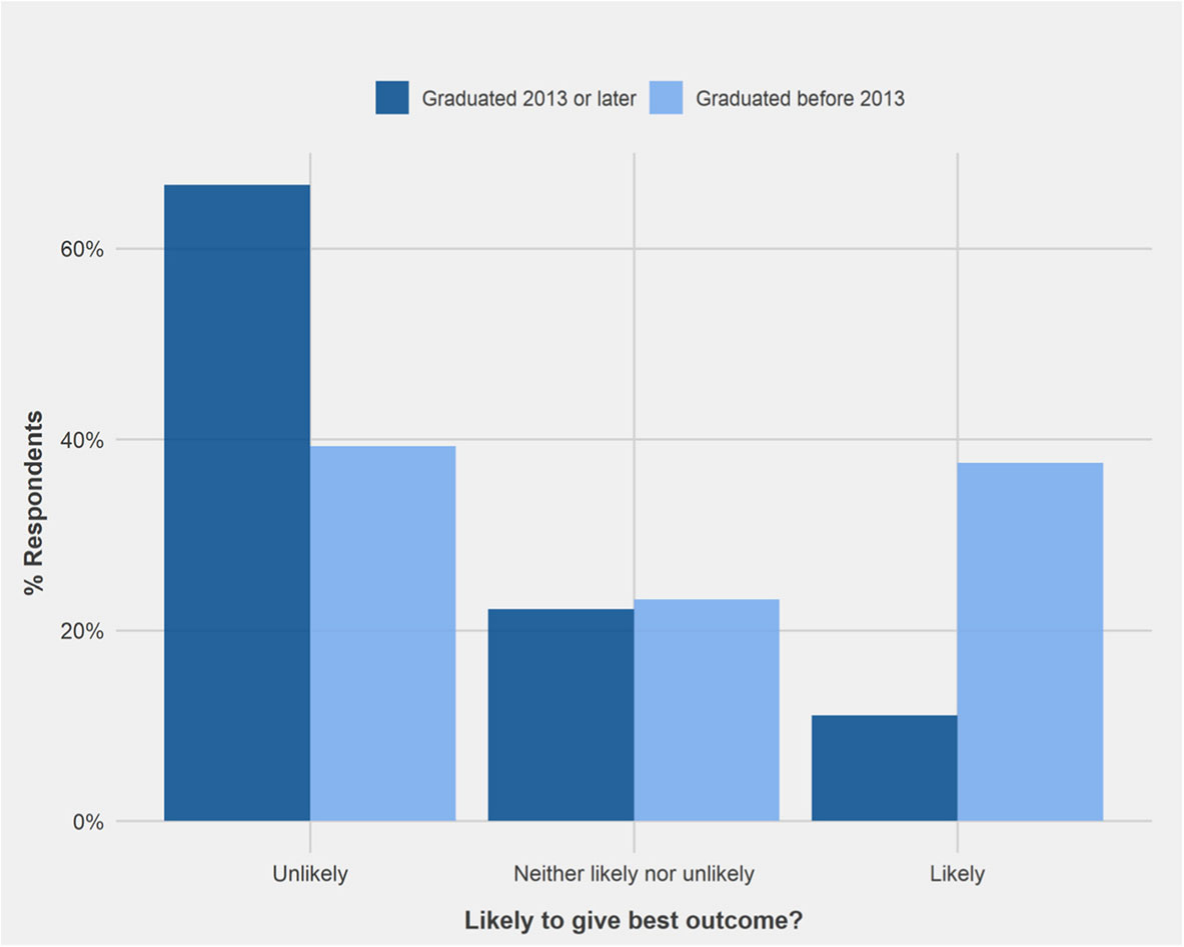
Comparison of the responses of Private Veterinary Practitioners who graduated 2013 or later (*n* = 28) and those who graduated before 2013 (*n* = 56) regarding the likelihood of advice to support best outcome for the dog in Vignette 8: ‘Use of invisible radio fence to prevent wandering’

PVPs who graduated since 2013 responded to vignette 8: ‘invisible radio fence’ significantly differently to PVPs who graduated before 2013 (W = 1006.5, *p* < 0.05). Sixty- 7 % of PVPs who graduated since 2013 considered that the advice to use an invisible fence was unlikely to achieve best outcome for the dog, compared to only 39.3% of those who graduated before 2013. In addition, only 11.1% of PVPs graduating since 2013 indicated that the advice was likely to support best outcome, in contrast to 37.5% of PVPs graduating before 2013 (Fig. 7).

**Fig. 8:**
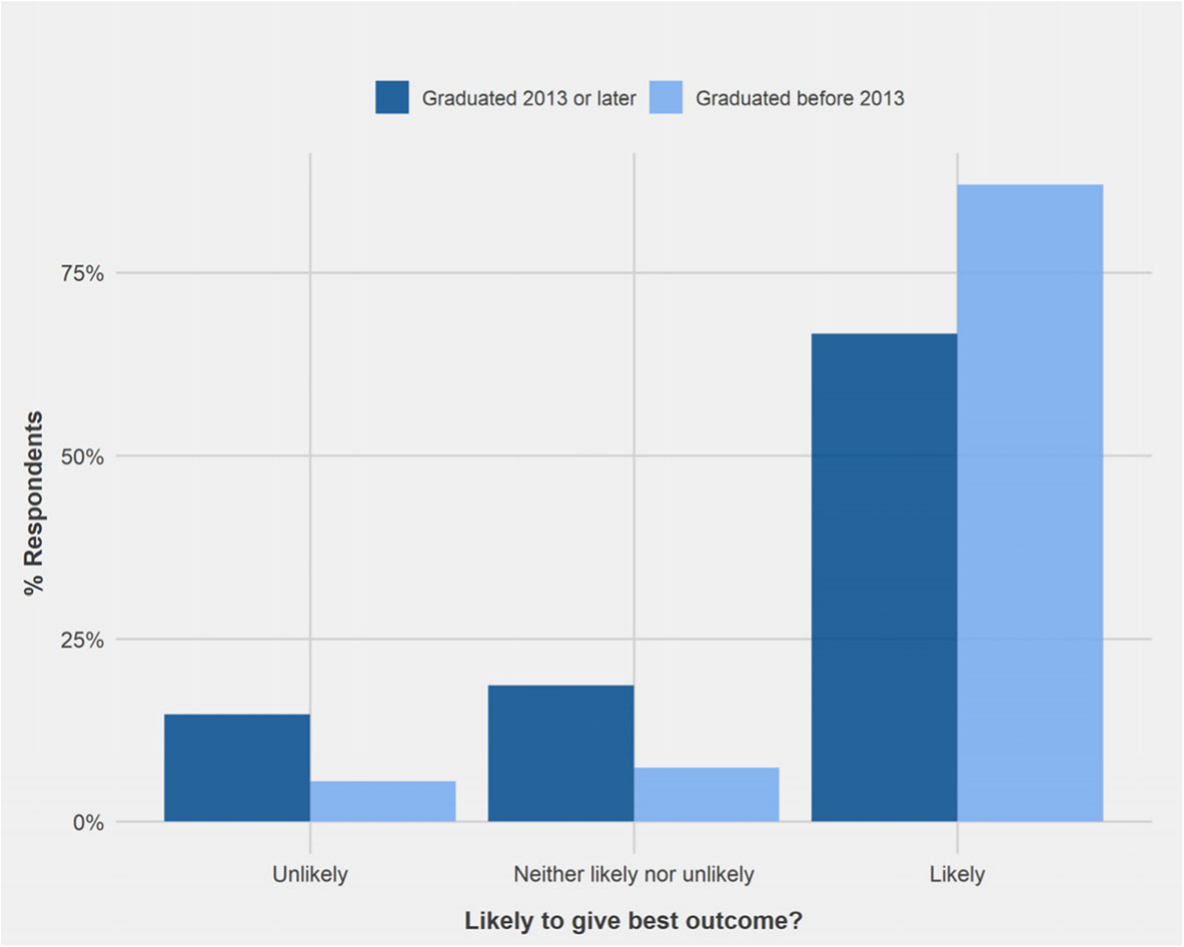
Comparison of the responses of Veterinary Nurses who graduated 2013 and later (n = 77) and those who graduated before 2013 (n = 56) regarding the likelihood of advice to support best outcome for the dog in Vignette 5: ‘Recommendation of reward-based accredited trainer for dog reactivity’

**Fig. 9:**
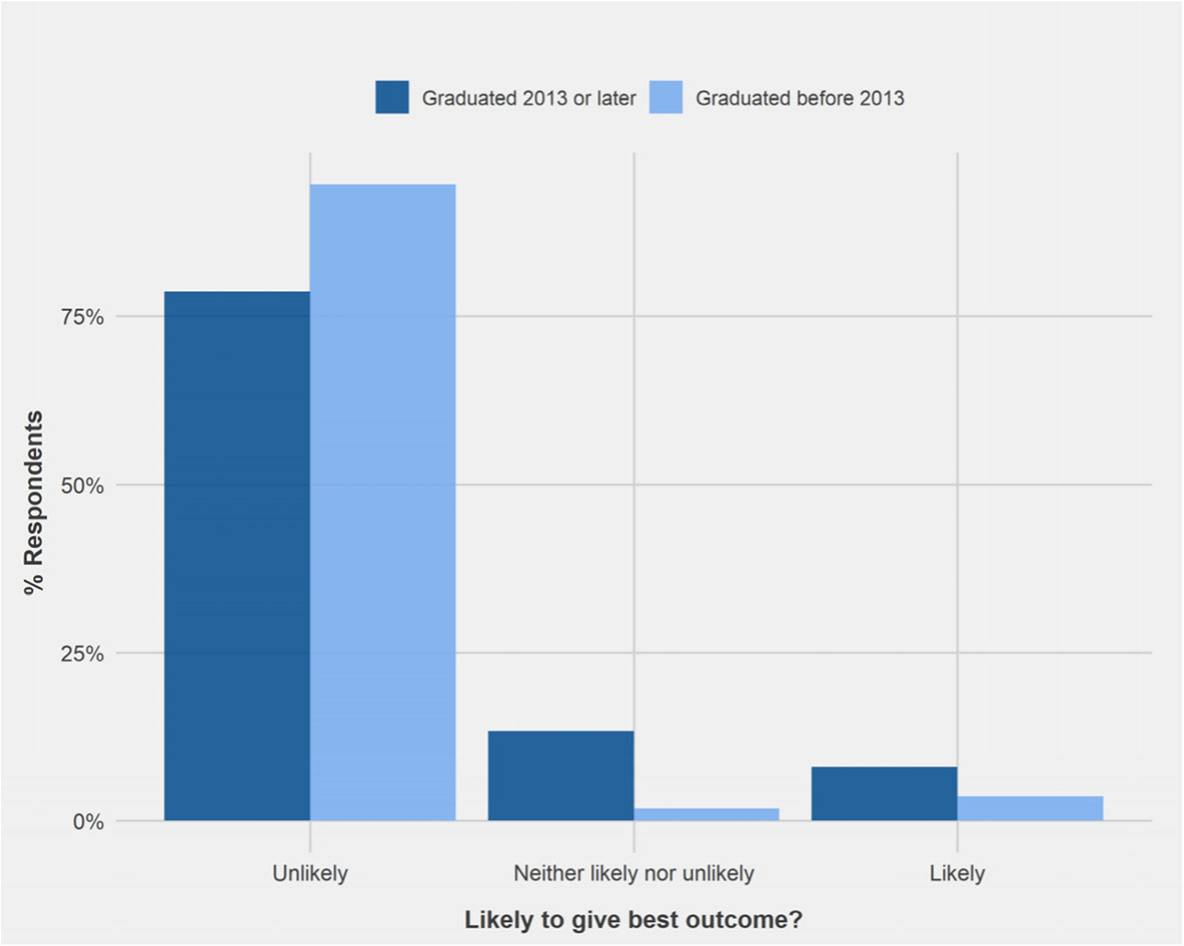
Comparison of the responses of Veterinary Nurses who graduated since 2013 (n = 77) and those who graduated before 2013 (n = 56) regarding the likelihood of advice to support best outcome for the dog in Vignette 11 ‘Use of desensitisation to treat fear behaviour’

VNs who graduated since 2013 responded to vignette 5: ‘accredited trainer’ and vignette 11 ‘flooding’ significantly differently than VNs who graduated before 2013 (W = 2590.5, p < 0.05; W = 1818.0, p < 0.05). The majority of VNs (87%) who graduated before 2013 recognised the likelihood of using an accredited trainer to support best outcome for the dog, compared to 66.7% of those graduated since 2013 (Fig. 8). In a similar trend, 94.5% of VNs graduated before 2013 recognised that flooding was unlikely to achieve best outcome in vignette 11, compared to 78.7% of those graduating since 2013. In addition, 13.3% of those graduating since 2013 answered ‘Neither likely nor unlikely’, versus only 1.8% of those graduating before 2013 (Fig. 9).

### Comparison by Behavioural services offered

A Wilcoxon rank sum test was performed to compare responses of those who worked at practices which either did or did not offer behavioural services. No significant differences were found between the cohorts across all vignettes.

### Continuing veterinary education

The majority (93.5%) of respondents declared that they would like to learn more about veterinary behavioural medicine and the treatment of common companion animal behavioural problems. Regarding the format of future CVE, practical workshops using case studies of dog behaviour problems were most popular (87.6%), followed by theory based eLectures (61.2%), conferences (48.8%) and journal clubs (17.9%) (*n* = 201).

## Discussion

This survey aimed to explore veterinary professionals’ understanding of different approaches to training and treatment options for canine behaviour problems commonly encountered in practice, to identify areas where there exists a lack of understanding, and to help support the development of competences in veterinary behaviour medicine (VBM) in Ireland.

Gaining access to veterinary professionals in Ireland was a limiting factor. Due to data protection laws, links to the survey were distributed by third parties and via social media. The survey was accessed by 338 individuals, and completed by 217, comprising 84 PVPs and 133 VNs. Only participants identifying as a PVP or VN were permitted to complete the survey, as a result 37 individuals selecting ‘Other’ were automatically excluded from completing the survey. The response rate could not be calculated, as the method of distribution meant that the total number of people whom the survey reached could not be obtained.

### Demographics

The majority of respondents, both PVPs (39%) and VNs (68%), were recent graduates (2011 onwards), with a tendency for the PVP cohort to be older (33% graduating in/before 2000) than in VN respondents (5%). The better representation of newer graduates overall could be a result of the method of distribution, especially on social media, where demographics are likely to be younger.

### Behavioural services offered in veterinary practice

Survey respondents were asked to select the types of behaviour services offered in their practices. A similar percentage of respondents indicated that their practice offered at least one behaviour service, from the options of in-house behaviour consultations, puppy socialisation classes, training events, other and none. Those selecting ‘other’ were asked to specify the behaviour service. Respondents were not specifically asked whether they referred cases to a behaviour specialist, they were given the option of ‘other’ which provided an opportunity to specify that they referred cases to a behaviour specialist. However, respondents could have interpreted referring cases as an ‘other’ behaviour service, or as providing no behaviour service. Therefore, we are unable to accurately determine the percentage of respondents who indicated they offered no behaviour services but who may also have referred cases to a behaviour specialist.

### Vignette selection

Vignettes were constructed based on most common owner reported behaviour problems [21] and recent research from veterinary practice in Ireland [10]. Vignettes were devised to illustrate both advice that was either likely (three) or unlikely (twelve) to result in the best outcome for both treatment of the behaviour problem and welfare of the dog. In vignettes selected to illustrate advice unlikely to give the best outcome, the advice was constructed based on traditional approaches that are considered to be ineffective or harmful to the animal’s welfare. In some cases, the advice may result in resolution of the problem for a time, but also have negative implications for the welfare of the animal, and therefore respondents were asked to consider the best outcome scenario to be one which does not negatively impact the dog’s welfare.

Vignettes 1, 3, 4, 8 and 10 illustrate advice recommending positive punishment-based training techniques. Such training methods can have an undesirable effect on animal welfare [30]. Hiby et al., [14] found that the use of positive punishment-based methods for obedience training was less effective than training with rewards, and that it was associated with a number of problematic behaviours. In addition, positive punishment-based techniques can worsen aggression and lead to a risk for owners [4, 13, 33]. For these reasons, advice in the vignettes recommending positive punishment-based training techniques was considered unlikely to result in the best outcome. Conversely, in the vignettes illustrating the use of positive reinforcement in training (vignettes 2 and 7), the advice was considered likely to result in the best outcome.

The use of aversive training tools such as citronella collars, invisible radio fences and check chains are illustrated in vignettes 3, 8 and 10 respectively. Several studies have explored the efficacy of citronella collars for treating excessive barking, and these indicate that they are successful in the short-term, but eventually dogs become habituated to the device [19, 34]. Furthermore, use of these collars elicits stress responses in dogs [26], and while they may be considered a more humane alternative to electric shock collars by some, one study found no difference in stress in dogs wearing electric shock collars versus citronella spray collars [32]. Invisible radio fences make use of a shock delivered via an electric collar to keep a dog within a property’s boundary. Training with electric collars is controversial; their use can have detrimental effects on welfare [28], and a ban on their use is being implemented in the UK. The use of invisible radio fences has been tied to instances of human directed aggression [22], and their efficacy in confining dogs onto a property is questionable; Starinsky et al. [31] found that a greater percentage of dogs (44%) escaped when confined by an electronic than a physical fence (23.3%). The use of a check chain to correct pulling is illustrated in vignette 10; as previously mentioned, obedience training methods incorporating positive punishment are considered no more effective than training utilising rewards [14], and the use of check chains may have a negative impact on welfare, especially when utilised with excessive force [11]. Therefore, the use of these aversive training devices was considered unlikely to give the best outcome, in terms of long-term effectiveness and welfare of the dog.

Vignette 9 portrayed a PVP advising a local trainer using language based in dominance theory to help correct aggressive behaviour in a dog. In the past, dominance theory was often used to explain the human-dog relationship, though it has since been contested [5]. Proponents of this theory often advocate for physical corrections and other positive punishment-based training techniques, which can be dangerous for dogs and their owners, as it may lead to, or worsen, aggressive behaviour [13]. For this reason, the recommendation of a trainer using dominance language was considered unlikely to support the best outcome. In contrast, the VN in vignette 5 recommends a trainer from the Association of Pet Dog Trainers (APDT) Ireland, accredited dog trainers who use reward-based methods. Here the advice was considered likely to result in the best outcome due to the accreditation, codes of the practitioner organisation and methods of the trainer [18].

Vignettes 6, 11 and 12 illustrate scenarios in which advice is given based on misunderstanding of learning theory, or where no evidence exists supporting the advice. Vignette 6 portrays a VN advising an owner to comfort a puppy by petting it when it shows noise phobia. However, this advice is likely to result in positive reinforcement of the fear behaviour [29]. In vignette 11, a PVP advises an owner with a puppy displaying a fear of children to get the puppy handled by as many children as possible. This advice is an example of flooding, and is likely to result in undesired effects on the socialisation process from excessive stress [3, 8]. Vignette 12 portrays advice from a PVP based on a commonly held misconception, that acquiring another dog will help treat separation anxiety by providing companionship. Several studies have found that having multiple dogs in the home does not protect against separation anxiety [12, 20], and it is possible that social transmission could lead to a newly acquired dog also beginning to exhibit signs of separation anxiety. Indeed, this vignette illustrates the complexity of behaviour cases and the need for veterinary professionals to be educated further. The vignette described destructive behaviour that the dog performed when alone. As with aggressive behaviour, there are several possible reasons why this can occur, one of which may be separation anxiety [1].

### Identification of advice to support best outcome for dog welfare by veterinary professionals

The survey is instructive, as it highlights areas of misunderstanding regarding the application of learning theory and best practice advice for common dog behaviour problems. In the majority of vignettes, over 50% of PVPs and VNs agreed with our classification of best outcome. However, there were several vignettes where areas of uncertainty were identified for PVPs (vignettes 3, 8, 9 and 12) and VNs (vignettes 9 and 12). There was an inconsistency in the understanding of positive punishment-based training, whilst the vast majority of PVPs recognised the unlikelihood that it would lead to best outcome in the case of the check chain (vignette 10), they were less clear about the use of the citronella collar and invisible fence (vignettes 3 and 8). It may be that respondents considered the citronella collar to be a more humane alternative to electronic bark collars, as 19% indicated they thought the advice was likely to result in best outcome, despite evidence indicating similar stress levels in both collars [32]. However, 8% also indicated that they did not know whether it would provide the best outcome; this vignette represented the second highest percentage of ‘don’t know’ responses in the survey, and the variation in response may reflect a lack of knowledge about citronella collars.

Despite concerns about the risks to dog welfare relating to the use of invisible radio fences, almost one third of PVPs responded that their use would likely give the best outcome, with very few (1%) answering don’t know. This may reflect the context of sheep worrying and balancing the potential harms to both sheep and dog. Furthermore, the areas of uncertainly identified in vignettes 3 (citronella collar) and 8 (invisible radio fence) could represent the desire of a quick fix from the owner, and the need of the PVP to provide their client with a solution with which the client will follow through. Several respondents noted in the comments section of the vignettes that clients are often unwilling to implement advice. These devices could fulfil the desire for a quick fix by the owner whilst allowing the PVP to at least be able to offer some solution to rectify a problem which could potentially lead to rehoming or euthanasia of the animal (especially in cases of sheep worrying). A report from the UK suggests that most cases of livestock worrying occur when the dog is left unsupervised outdoors, unaccompanied by the owner or off-leash during walks [17]. Therefore, failure of effective confinement when dogs are left unsupervised is a key risk factor for sheep worrying. As previously discussed, electronic fences are less likely to prevent escape than physical fences [31]. In addition, the stress elicited by the use of these aversive training devices could potentially lead to the manifestation of further behaviour problems in the future.

Another area of uncertainty from veterinary professionals related to vignette 9 (dominance trainer). Several respondents who indicated that this scenario was unlikely to support best outcome commented that they understood that dominance theory and positive punishment-based methods used by the trainer could result in worsening of the aggression. In contrast, 27% of PVPs and 36% of VNs indicated that it was likely to support the best outcome for the dog. Such differences may reflect either a failure to recognise the language used in the vignette as being associated with dominance theory and thus positive punishment-based methods, or support for this method of training. Several respondents (20% of PVPs, 17% of VNs) answered ‘neither likely nor unlikely’ in this vignette, with some commenting that they would require more details on the trainer to make a conclusion. While not directly recognising the use of language implicating dominance theory-based methods, this still indicates that these respondents recognise that the methods of a trainer must be investigated before making a referral.

Over the last 20 years there has been an increasing awareness of providing stimulation for companion dogs, and not leaving them in the house on their own all day. It is also a commonly held belief that acquiring another dog is appropriate to address separation anxiety, as illustrated in vignette 12. This advice proved to be an area of uncertainty for both PVPs and VNs, with only 39 and 44%, respectively agreeing with our classification of best outcome. As previously discussed, acquiring another dog is unlikely to treat separation anxiety [12, 20] yet over a quarter of PVPs (26%) and 21% of VNs responded that it was likely to give the best outcome. This finding is concerning, given the relatively high incidence of anxiety related behaviours encountered in practice [10]. The PDSA [21] reported that 31% of owners sought their veterinarian’s advice if they encountered behaviour problems, and this finding underpins the need for undergraduate training and CVE on veterinary behavioural medicine to support evidence-based advice to clients.

The vignette with the highest percentage of PVPs responding ‘don’t know’ was vignette 5 (10% vs 3% of VNs), advocating referral to an accredited trainer to help with dog reactivity. Comments from some respondents reflect a failure to recognise the accredited organisation, APDT. Since consultations do not always provide sufficient time to address behaviour problems, referral to other sources, such as behaviourists or trainers, is often necessary. There are no official requirements to qualify and practice as a dog trainer or behaviourist in Ireland. It is important that veterinary professionals be informed of the credentials of individuals to who they refer clients, to ensure that the methods used correspond to best practice. In addition, less VNs who graduated since 2013 recognised the likelihood of using an accredited trainer to support best outcome for the dog in this vignette compared with VNs who graduated before 2013. Possible explanations include that more recent VN graduates may not be as aware of the need for accreditation or that VNs graduating before 2013 have spent longer in practice and have had more opportunity to become familiar with the different accreditations used.

Furthermore, some respondents indicated that owners are often either unwilling to follow through with a trainer, referral or unreceptive to unsolicited advice. Roshier and McBride [24] found that while discussion of medical problems in consultations was largely instigated by PVPs, discussions around behaviour problems were instigated equally by both PVPs and clients, and that for many behaviour cases, they were not explored further. If veterinary professionals lack confidence in their ability to advise about behaviour problems and effectively communicate recommendations, as indicated in other research [10, 25], then they may be less willing to address issues of behaviour. This further supports the need for improved undergraduate training and CVE in VBM.

### Comparison by profession

Generally, the same patterns of responses were recorded for PVPs and VNs. An exception was for the use of aversive training devices in vignette 3 (citronella collar) and 8 (invisible radio fence). In both cases more PVPs than VNs responded that it was likely to support the best outcome and conversely more VNs than PVPs responded that they were unlikely to achieve the best outcome. This may be reflected by differences in undergraduate training, for example, at UCD the VN programme has a greater focus on animal behaviour and welfare than the veterinary medicine programme. Alternatively, it may be indicative of demographic differences (i.e. year of graduation) in respondents.

### Comparison by graduation date

Significantly more PVPs graduating since 2013 (67%) recognised that use of an invisible radio fence (vignette 8) would be unlikely to result in the best outcome than earlier graduates (39.3%). At UCD 2013 represents the first year when graduates were exposed to Bradshaw et al. [5] during their preclinical training; as a result, newer graduates may be less accepting of training methods relying on positive punishment, which is often advocated for by proponents of dominance theory. However, this does not explain the lack of difference recorded for other positive punishment-based training techniques.

The results obtained for VNs graduating before and since 2013 are more difficult to explain. Interestingly, a far higher percentage of earlier graduates were able to identify that best outcome was likely using an accredited trainer (vignette 5) and unlikely that using flooding to treat fear (vignette 11) than newer graduates. The BSc in Veterinary Nursing degree was first offered at UCD from 2009, with first graduates in 2013, so these differences could reflect changes to the course. However, data was not collected on where VNs received their training. As such, these findings may reflect the diversity of VN courses offered in Ireland and not as a result of specific course changes. In addition, these results reflect a need for standardisation of teaching in VBM across veterinary education, such as identifying day one competences.

### Continuing veterinary education

Participants indicated a strong desire to learn more about VBM through CVE. In the survey participants were requested to identify their prefered learning format selecting from workshops using case studies, theory based eLectures, conferences and journal clubs. The majority identified workshops, probably because case studies provide a real-life practical context to addressing behaviour problems. One disadvantage of case studies is that they are case-specific and reflect the decision-making process of a single practitioner. However, in a workshop environment they also provide a basis for discussion, to explore other treatment options.

eLectures were identified as a means to address theory-based learning. Online learning offers a flexible learning environment for PVPs and VNs working in practice. The least selected option was journal clubs.

## Conclusions and recommendations

Overall, the most frequent response of both PVPs and VNs in each vignette corresponded with our classification of best outcome. This indicates that the majority of veterinary professionals have a good understanding of the training techniques and behavioural theory backed by current literature. However, several vignettes were identified with high levels of disagreement, indicating that there may be lack of knowledge in specific areas, especially surrounding the use of positive punishment-based training and aversive training devices. This could reflect the presence of superficial learning, as there appears to be good understanding of the application of operant conditioning in some areas but not others. These findings indicate that some traditional approaches to behaviour modification persist in the veterinary profession and imply that in newer graduates, at least in PVPs, there is a trend towards support of reward-based methods over positive punishment-based methods. These findings have important implications for curriculum design such as the development of day one competences in VBM.

Veterinary behaviour medicine is essential to companion animal welfare. This study has identified potential to further develop behavioural services offered in veterinary practice or via referral and to ensure that dog trainers and behaviourists used for referrals by PVPs are accredited and belong to organisations with an appropriate code of conduct regarding training methods. Respondents overwhelmingly indicated a desire to learn more about VBM, and the findings of this survey convey the need for development of CVE. Furthermore, our research highlights specific areas of uncertainty regarding veterinary professionals’ knowledge of animal behaviour and welfare, which provides further evidence to support the development of day one competences in VBM for veterinary medicine and veterinary nursing programmes at university level.

## Additional file

Additional file 1: PVP_VN Online Survey. (PDF 101 kb)
