# Supplementary material for: Exploring the understanding of best practice approaches to common dog behaviour problems by veterinary professionals in Ireland
Source: Ir Vet J. 2019 Mar 21;72:1. doi: 10.1186/s13620-019-0139-3 (PMC6429707; doi:10.1186/s13620-019-0139-3)
Supplement: Supplementary file 1 — PVP_VN Online Survey. (PDF 101 kb) [file 13620_2019_139_MOESM1_ESM.pdf]

Welcome

Dear Veterinary Professional,

I am a graduate entry veterinary student at UCD conducting a summer research project in conjunction with Associate Professor Alison Hanlon and Mark McCorry RVN BScAAB. The project explores veterinary professionals' approach to canine behavioural problems and is supported by the Interchem Summer Student Research Award 2018.

We would really appreciate your input to help us gain valuable insight into the current understanding of veterinary professionals in Ireland about treatment options for frequently encountered behavioural problems. This will help to support improvements in veterinary education in Ireland and the development of competences in veterinary behavioural medicine.

The survey will take less than 10 minutes to complete. It consists of three sections: Practice role and experience (4 questions), common behaviour scenarios (12 questions) and outlooks on continuing education (2 questions). No identifiable data is requested and so responses are anonymous. The survey closes on Tuesday the 7th of August.

We are grateful for your participation and appreciate your time.

Sincerely,
Emma Shalvey

emma.shalvey@ucdconnect.ie

Consent

1. I give my consent to participating in this project and understand that the data will be used for research purposes and to support the development of veterinary education and competences in veterinary behaviour medicine.

Agree/Disagree

Section 1: Professional role and Experience

2. Are you:
   Private Veterinary Practitioner ☐
   Veterinary Nurse ☐
   Other (please specify)

3. What year did you graduate?

4. What behavioural services are offered in your practice? Tick all that apply
   Puppy socialisation classes ☐
   Training events ☐
   In-house behaviour consultations ☐
   None ☐
   Other (Please specify)

Section 2: Scenarios on Common Canine Behavioural Problems

The following section contains scenarios on common dog behavioural problems that you may encounter in practice. You will be asked to consider how likely the veterinary professional's recommendation is to give the best outcome in each given scenario. **The best outcome is defined as one which provides a resolution to the behavioural problem while not compromising the animal's welfare.**

5. Sarah has brought in her 1-year-old Labrador cross Toby to the vet for his annual check-up. She asks how to stop Toby from jumping up and mouthing, "*He's knocked the kids over several times!*"
   The vet tells Sarah, "*Try pushing Toby down and saying 'STOP' when he jumps up and mouths, to discourage the behaviour''*.

   How likely is this recommendation to give the best outcome?
   Extremely likely ☐ Likely ☐ Neither likely nor unlikely ☐ Unlikely ☐Extremely unlikely ☐ Don't know ☐

   Comments on your experience of this type of scenario **(optional):**

6. Paul has brought in his 8-week-old puppy for its vaccinations. He asks Sinead, the vet nurse, how to toilet train his new puppy. Sinead advises, "*Take the puppy outside at regular intervals and praise him whenever he toilets outside and don't punish him if he has an accident inside – but make sure to clean it up properly!*" She gives Paul a leaflet on the ''do's and don'ts'' of toilet training a new puppy.

   How likely is this recommendation to give the best outcome?
   Extremely likely ☐ Likely ☐ Neither likely nor unlikely ☐ Unlikely ☐Extremely unlikely ☐
   Don't know ☐

   Comments on your experience of this type of scenario **(optional):**

7. The neighbours have complained about Louise's two dogs that bark excessively while she is at work. While buying food at the vets, she asks Lauri, the vet nurse, for advice. Lauri suggests using anti-bark spray collars, *"They give the dog a warning beep before spraying citronella, they don't harm the dog at all".*

How likely is this recommendation to give the best outcome?
Extremely likely ☐ Likely ☐ Neither likely nor unlikely ☐ Unlikely ☐ Extremely unlikely ☐
Don't know ☐

Comments on your experience of this type of scenario **(optional):**

8. Jack's dog Monty is terrified of getting his nails clipped. He asks Val, a vet nurse, for advice to reduce Monty's fear. Val advises, "*No dog likes getting their nails done, you've just got to restrain them and push through or else they will learn to get away with it*".

How likely is this recommendation to give the best outcome?
Extremely likely ☐ Likely ☐ Neither likely nor unlikely ☐ Unlikely ☐ Extremely unlikely ☐
Don't know ☐

Comments on your experience of this type of scenario **(optional):**

9. Stephen's 4-year-old Husky lunges, growls and barks at other dogs in the vet clinic waiting room. Suzy, a vet nurse, notices the difficulty Stephen is having with controlling his dog and offers Stephen a business card, "*Several clients have had help from one of our registered APDT Ireland trainers*".

How likely is this recommendation to give the best outcome?
Extremely likely ☐ Likely ☐ Neither likely nor unlikely ☐ Unlikely ☐ Extremely unlikely ☐
Don't know ☐

Comments on your experience of this type of scenario **(optional):**

10. Lucy has brought her 5-month-old puppy in to the vets for a quick weigh-in. Lucy asks the vet nurse, Chris, if he has any recommendations to help with her puppy's fear during fireworks, "*She hides behind the couch all night when they're going"* Chris offers her advice "*Give her lots of cuddles and praise when she's feeling scared to help her feel more comfortable*".

How likely is this recommendation to give the best outcome?
Extremely likely ☐ Likely ☐ Neither likely nor unlikely ☐ Unlikely ☐Extremely unlikely ☐
Don't know ☐

Comments on your experience of this type of scenario **(optional):**

11. George has brought in his Jack Russell Terrier, Skip, for a check-up following surgery after a road traffic accident. Shannon, the vet, gives Skip the all clear, but George is worried about letting him off lead again because Skip normally runs off and ignores his calls.
Shannon suggests a local obedience class, "*Give these classes a try and keep Skip on a long lead during walks until you're comfortable he'll come back to you. If you regularly call him during your walks and reward him for coming back he'll start to get the idea''.*

How likely is this recommendation to give the best outcome?
Extremely likely ☐ Likely ☐ Neither likely nor unlikely ☐ Unlikely ☐Extremely unlikely ☐
Don't know ☐

Comments on your experience of this type of scenario **(optional):**

12. John's German Shepherd, Max, is repeatedly escaping from the garden. There have been recent cases of sheep worrying in the area and he's looking for advice from the local practice. The vet suggests, "*It depends on how much time and money you are willing to invest – the quickest way is to install an invisible radio fence*".

How likely is this recommendation to give the best outcome?
Extremely likely ☐ Likely ☐ Neither likely nor unlikely ☐ Unlikely ☐Extremely unlikely ☐
Don't know ☐

Comments on your experience of this type of scenario **(optional):**

13. During a routine clinical examination, Greta's Border Collie cross Lulu snaps at the vet. Greta admits that Lulu can be aggressive, especially towards strangers whilst on walks. The vet recommends a local trainer and tells her, "*This guy knows his stuff and will set her straight, she needs to learn you're in charge otherwise she will keep trying to protect you and hurt other people*".

How likely is this recommendation to give the best outcome?
Extremely likely ☐ Likely ☐ Neither likely nor unlikely ☐ Unlikely ☐Extremely unlikely ☐
Don't know ☐

Comments on your experience of this type of scenario **(optional):**

14. On arrival at the vet clinic, Julie almost falls over as Ben, her Saint Bernard, pulls her through the door. The vet nurse at the desk sees that Julie is having problems controlling Ben and says, "*Have you considered using a check chain as a training aid? It's the best way to control a big dog like Ben*".

How likely is this recommendation to give the best outcome?
Extremely likely ☐ Likely ☐ Neither likely nor unlikely ☐ Unlikely ☐Extremely unlikely ☐
Don't know ☐

Comments on your experience of this type of scenario **(optional):**

15. Tom has brought his new puppy, Penny, to the vets for her first vaccinations, "*She's terrified of the kids at home and just cowers in the corner"*
The vet replies, *"Get as many kids in from the neighbourhood as possible to handle her, that should get her well socialised''*.

How likely is this recommendation to give the best outcome?
Extremely likely ☐ Likely ☐ Neither likely nor unlikely ☐ Unlikely ☐Extremely unlikely ☐
Don't know ☐

Comments on your experience of this type of scenario **(optional):**

16. Emily has brought her lurcher, Finn, to the vet to get treatment for an injured paw after he attempted to escape from his crate "*I feel awful, but I have to confine him in there when I'm at work otherwise he destroys the house, he gets so distressed when I leave''*
Fran, the vet, has seen this problem many times and suggests, *"Have you considered getting another dog to keep Finn company?"*

How likely is this recommendation to give the best outcome?

Extremely likely ☐ Likely ☐ Neither likely nor unlikely ☐ Unlikely ☐ Extremely unlikely ☐
Don't know ☐

Comments on your experience of this type of scenario **(optional):**

Section 3. Continuing Education

17. Would you like to learn more about veterinary behavioural medicine and the treatment of common companion animal behavioural problems?
    Yes ☐
    No ☐

18. What kind of CVE in companion animal behaviour would you like to see offered in the future? Tick all that apply
    Theory based eLectures ☐
    Practical workshop using case studies of dog behaviour problems ☐
    Journal club ☐
    Conferences ☐
    Other (Please specify)
